# New acetylenic derivatives of betulin and betulone, synthesis and cytotoxic activity

**DOI:** 10.1007/s00044-016-1713-9

**Published:** 2016-09-01

**Authors:** Ewa Bębenek, Monika Kadela-Tomanek, Elwira Chrobak, Joanna Wietrzyk, Joanna Sadowska, Stanisław Boryczka

**Affiliations:** 1 Department of Organic Chemistry, Medical University of Silesia in Katowice, School of Pharmacy with the Division of Laboratory Medicine in Sosnowiec, 4 Jagiellońska Str., Sosnowiec, 41-200 Poland; 2 Department of Experimental Oncology, Polish Academy of Sciences, Ludwik Hirszfeld Institute of Immunology and Experimental Therapy, 12 R. Weigla Str., Wrocław, 53-114 Poland

**Keywords:** Betulin, Betulone, Synthesis, Cytotoxic activity, Lipophilicity

## Abstract

Betulin **1** and its semisynthetic derivatives exhibit a cytotoxic activity toward various cancer cell lines. These compounds are a promising and potential anticancer candidates. A series of betulin derivatives was prepared and tested for the antiproliferative activity in vitro against T47D breast cancer, CCRF/CEM leukemia, HL-60 promyelocytic leukemia, SW707 colorectal, murine P388 leukemia, as well as BALB3T3 normal fibroblasts cell lines. Cisplatin and betulin **1** were used as a reference compounds. Some derivatives of betulin showed a higher cytotoxic activity than the parent compound **1**. Two derivatives (**5** and **17**) were 24-fold potent than betulin **1** against the human promyelocytic leukemia cell line (HL-60), with an IC_50_ value of 0.3 µg/mL.

## Introduction

**Fig. 1:**
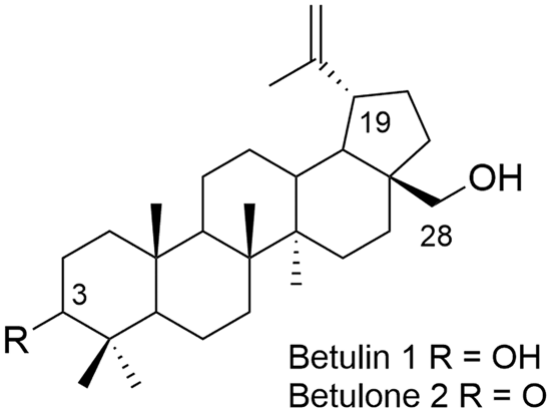
Chemical structure of betulin **1** and betulone **2**

Betulin (lup-20(29)-ene-3β,28-diol) **1** is a pentacyclic triterpene of the lupane type which is isolated from bark of white birch species (Fig. 1). The compound **1** has three active positions in its structure, namely the primary hydroxyl group at C-28, the secondary group at C-3 and the isopropenyl side chain at C-19. It’s possible to make a chemical modification of these positions to obtain new betulin derivatives with important biological properties such as antitumor, antiviral, antimicrobial, anti-inflammatory, as well as hepatoprotective activities (Alakurtti et al., 2006; Tolstikov et al., 2005).

Betulone (lup-20(29)-en-28-ol-3-one) **2** similarly to betulin **1** represents a class of pentacyclic triterpenes which can be isolated from various plants (Fig. 1) (Diouf et al., 2009; Liu et al., 2012; Reyes et al., 2006). The content of betulone **2** in the native plant material is very low, therefore the compound **2** was obtained by oxidation or biotransformation of naturally occurring betulin **1** (Grishko et al., 2013; Hase et al., 1981; Mao et al., 2012). Despite the fact that betulone **2** has been isolated from *Betula lenta* in 1991 (Cole et al., 1991), the crystal structure of this compound was determined for the first time in 2013 (Boryczka et al., 2013a).

It has already been reported that betulone **2** possess interesting pharmacological activities such as anti-leishmanial, anti-inflammatory, and aniparasitic against *Plasmodium falciparum* and *Trypanosoma brucei rhodesiense* (Alakurtti et al., 2010; Gachet et al., 2011; Reyes et al., 2006). Triterpene **2** exhibited also antifouling activity against cyprid larvae of the barnacle *Balanus albicostatus* with the EC_50_ value 8.73 µg/mL slightly higher than betulin **1** (Chen et al., 2011). The compound **2** demonstrated almost the same protective effects as betulin **1** against the cytotoxicity of cadmium at high concentrations (Hiroya et al., 2002). Betulone **2** with the carbonyl group at C-3 position showed anticancer effect on mouse melanoma (B16 2F2) cell line with the IC_50_ value 29.3 µM (Hata et al., 2002). Additionally, the compound **2** and its derivatives showed in vitro cytotoxic activity against different cancer cell lines like stomach (MGC-803), breast (Bcap-37, MCF-7), prostate (PC3), melanoma (SK-MEL-2, A-375), medulloblastoma (Dayo), glioblastoma (LN-229), ovarian carcinoma (OVCAR-3), and colon carcinoma (HT-29) (Koohang et al., 2009; Liu et al., 2012; Mar et al., 2009). Derivatives of betulone containing 3′-substituted glutaryl groups at C-28 position represent a new class of anti-HIV agents. These compounds exhibited anti-HIV activity with EC_50_ values in the range of 4.3–10.0 µM (Sun et al., 1998a; Sun et al., 1998b).

We have previously described the synthesis and evaluation of cytotoxicity of betulin derivatives containing one or two acetylenic groups at the C-3 and/or C-28 positions. Our studies showed, that the derivative of betulin with a propynoyl group at C-28 position, has strong cytotoxic effects against human leukemia (CCRF/CEM) and murine leukemia (P388) cancer cells. Moreover, 28-*O*-propynoylbetulin induces apoptosis in human melanoma (G-361) cells via caspase-3 activation (Boryczka et al., 2013b; Orchel et al., 2014).

Continuing our research project on the development of anticancer agents, we synthesized new compounds, in order to obtain more information about the influence of alkane, alkene, and alkyne moiety at the C-28 position on antiproliferative activity in the group of betulin and betulone derivatives.

## Materials and methods

### Chemistry

Melting points of betulin derivatives were obtained in open capillary tubes on a Boetius melting point apparatus without correction. Nuclear magnetic resonance (NMR) (600/150 MHz) spectra were measured in CDCl_3_ as solvent on a Bruker Avance III 600 spectrometer. The chemical shifts values are reported in ppm (*δ*) and the coupling constants (*J*) are presented in Hertz. The spin multiplicities are given as singlet (s), doublet (d), triplet (t), q (quartet), and multiplet (m). Mass spectra were measured under EI conditions on a Finnigan MAT 95 spectrometer. Infrared spectra (KBr, pellet) were recorded using the IRAffinity-1 Shimadzu spectrometer and reported in wave number (cm^−1^). The progress of all reactions were monitored by thin layer chromatography (TLC) on silica gel 60 254F plates using a mixture of chloroform and ethanol (40:1, v/v) as an eluent. The spots were visualized by spraying with a solution of 5 % sulfuric acid and then heating to 100 °C. Purification of the new compounds was carried out by column chromatography (silica gel 60, <63 μm, Merk) using a mixture of chloroform and ethanol (40:1, v/v) as an eluent. All solvents for reactions were dried and purified prior to use.

The synthesis and spectral data of the compounds **3**, **5**, **7**, **12**–**14** was described previously by Boryczka et al. (2013b).

#### General procedure for the synthesis of derivatives **4**, **6**, **8**–**9**

To a mixture of betulin **1** (0.44 g, 1 mmol) and 2-propenoic acid (0.08 g, 1.10 mmol), 3-cyclopropyl-2-propynoic acid (0.12 g, 1.10 mmol), 2-butenoic acid (0.09 g, 1.10 mmol) or 2-butynoic acid (0.9 g, 1.10 mmol) in dichloromethane (5 mL) was added slowly a solution of dicyclohexylcarbodiimide (0.23 g, 1.12 mmol), and 4-dimethylaminopyridine (0.01 g, 0.08 mmol) in dichloromethane (1 mL) at −10 °C temperature. The reaction was stirred under argon atmosphere at −10 °C temperature for 5 h, and then was allowed to warm to room temperature and stirred overnight. The progress of the reaction was monitored by TLC until completion and then filtered. The solvent was removed under reduced pressure and the residue was purified by silica gel column chromatography (chloroform/ethanol 40:1, v/v).

28-*O*-(2-Propenoyl)betulin (**4**) Yield 74 %; mp 117–121 °C; *R*_f_ 0.44 (chloroform/ethanol, 40:1, v/v); IR (KBr) *ν*_max_ 3445, 2939, 1723, 1456, 1269 cm^−1^; ^1^H NMR (600 MHz, CDCl_3_): *δ* 6.42 (1H, m, CH=CH_2_), 6.15 (1H, m, CH=CH_2_), 5.84 (1H, m, CH=CH_2_), 4.71 (1H, s, H-29), 4.61 (1H, s, H-29), 4.36 (1H, d, *J* = 10.8 Hz, H-28), 3.95 (1H, d, *J* = 10.8 Hz, H-28), 3.20 (1H, m, H-3), 2.49 (1H, m, H-19), 1.67 (3H, s, CH_3_), 1.06 (3H, s, CH_3_), 0.98 (3H, s, CH_3_), 0.96 (3H, s, CH_3_), 0.84 (3H, s, CH_3_), 0.77 (3H, s, CH_3_); ^13^C NMR (150 MHz, CDCl_3_): *δ* 166.7 (O–C=O), 150.2 (C-20), 130.5, 128.6, 109.9 (C-29), 79.0 (C-3), 62.8 (C-28), 55.3, 50.4, 48.8, 47.7, 46.5, 42.7, 40.9, 38.9, 38.7, 37.6, 37.1, 34.6, 34.2, 29.8, 29.6, 28.0, 27.4, 27.1, 25.2, 20.8, 19.1, 18.3, 16.1, 16.0, 15.4, 14.8; EIMS *m/z* 496 [M]^+^ (14), 189 (100).

28-*O*-(3-Cyclopropyl-2-propynoyl)betulin (**6**) Yield 49 %; mp 108–113 °C; *R*_f_ 0.44 (chloroform/ethanol, 40:1, v/v); IR (KBr) *ν*_max_ 3475, 2941, 2228, 1707, 1454, 1258 cm^−1^; ^1^H NMR (600 MHz, CDCl_3_): *δ* 4.68 (1H, s, H-29), 4.58 (1H, s, H-29), 4.31 (1H, d, *J* = 10.8 Hz, H-28), 3.93 (1H, d, *J* = 10.8 Hz, H-28), 3.18 (1H, m, H-3), 2.42 (1H, m, H-19), 1.67 (3H, s, CH_3_), 1.01 (3H, s, CH_3_), 0.97 (3H, s, CH_3_), 0.96 (3H, s, CH_3_), 0.92–0.91 (5H, m, CH, CH_2_), 0.82 (3H, s, CH_3_), 0.77 (3H, s, CH_3_); ^13^C NMR (150 MHz, CDCl_3_): *δ* 154.5 (O–C=O), 150.1 (C-20), 109.9 (C-29), 93.3, 78.9 (C-3), 68.6 (C-28), 64.1, 55.3, 50.4, 48.8, 47.7, 46.4, 42.7, 40.9, 38.9, 38.7, 37.6, 37.1, 34.5, 34.2, 29.7, 29.5, 28.0, 27.4, 27.0, 25.2, 20.8, 19.1, 18.3, 16.1, 16.0, 15.3, 14.8, 9.2, 1.1, -0.6; EIMS *m/z* 534 [M]^+^ (18), 189 (100).

28-*O*-(2-Butenoyl)betulin (**8**) Yield 57 %; mp 182–184 °C; R_f_ 0.45 (chloroform/ethanol, 40:1, v/v); IR (KBr) *ν*_max_ 3560, 2945, 1707, 1443, 1195 cm^−1^; ^1^H NMR (600 MHz, CDCl_3_): *δ* 7.00 (1H, m, CH=CHCH_3_), 5.88 (1H, m, CH=CHCH_3_), 4.72 (1H, s, H-29), 4.61 (1H, s, H-29), 4.34 (1H, d, *J* = 10.8 Hz, H-28), 3.93 (1H, d, *J* = 10.8 Hz, H-28), 3.21 (1H, m, H-3), 2.48 (1H, m, H-19), 1.91 (3H, m, CH=CHCH_3_), 1.68 (3H, s, CH_3_), 1.06 (3H, s, CH_3_), 1.00 (3H, s, CH_3_), 0.99 (3H, s, CH_3_), 0.85 (3H, s, CH_3_), 0.71 (3H, s, CH_3_); ^13^C NMR (150 MHz, CDCl_3_): *δ* 167.0 (O–C=O), 150.2 (C-20), 144.4, 122.9, 109.8 (C-29), 78.9 (C-3), 62.4 (C-28), 55.3, 50.4, 48.9, 47.7, 46.5, 42.7, 40.9, 38.9, 38.7, 37.6, 37.2, 34.6, 34.2, 29.9, 29.7, 28.0, 27.4, 27.1, 25.2, 20.8, 19.2, 18.3, 16.1, 16.0, 15.4, 14.8, 3.7; EIMS *m/z* 510 [M]^+^ (14), 189 (100).

28-*O*-(2-Butynoyl)betulin (**9**) Yield 52 %; m.p. 111–113 °C; *R*_f_ 0.44 (chloroform/ethanol, 40:1, v/v); IR (KBr) *ν*_max_ 3482, 2942, 2245, 1709, 1457, 1248 cm^−1^; ^1^H NMR (600 MHz, CDCl_3_): *δ* 4.69 (1H, s, H-29), 4.59 (1H, s, H-29), 4.33 (1H, d, *J* = 10.8 Hz, H-28), 3.95 (1H, d, *J* = 10.8 Hz, H-28), 3.16 (1H, m, H-3), 2.42 (1H, m, H-19), 1.99 (3H, s, C≡CCH_3_), 1.68 (3H, s, CH_3_), 1.02 (3H, s, CH_3_), 0.98 (3H, s, CH_3_), 0.97 (3H, s, CH_3_), 0.82 (3H, s, CH_3_), 0.76 (3H, s, CH_3_); ^13^C NMR (150 MHz, CDCl_3_): *δ* 154.4 (O–C=O), 150.0 (C-20), 109.9 (C-29), 85.5, 79.0 (C-3), 72.5, 64.2 (C-28), 55.3, 50.4, 48.8, 47.6, 46.4, 42.7, 40.9, 38.9, 38.7, 37.6, 37.1, 34.5, 34.2, 29.7, 29.5, 28.0, 27.4, 27.0, 25.2, 20.8, 19.1, 18.3, 16.1, 16.0, 15.3, 14.8, 3.8; EIMS *m/z* 508 [M]^+^ (22), 189 (100).

#### General procedure for the synthesis of derivatives **10**–**11**

To a mixture of betulin **1** (0.44 g, 1 mmol) and pyridine (2.5 mL) in benzene (6 mL) at 0–5 °C temperature was added solution of propyl chloroformate or allyl chloroformate (3 mmol) in benzene (5 mL). The reaction was stirred at 0–5 °C temperature for 4 h. After this time the reaction was allowed to warm to room temperature and stirred overnight. The reaction mixture was diluted with 5 mL of chloroform and washed successively with 1 N sulfuric acid and water, then dried and concentrated under reduced pressure. The crude product was purified by silica gel column chromatography (chloroform/ethanol 40:1, v/v).

28-*O*-Propoxycarbonylbetulin (**10**) Yield 51 %. m.p. 92–95 °C; *R*_f_ 0.43 (chloroform/ethanol, 40:1, v/v); IR (KBr) *ν*_max_ 3536, 2941, 1743, 1457, 1267 cm^−1^; ^1^H NMR (600 MHz, CDCl_3_): *δ* 4.72 (1H, s, H-29), 4.61 (1H, s, H-29), 4.37 (1H, d, *J* = 10.8 Hz, H-28), 4.12 (2H, t, *J* = 6.6 Hz, OCH_2_), 3.94 (1H, d, *J *= 10.8 Hz, H-28), 3.20 (1H, m, H-3), 2.46 (1H, m, H-19), 1.73 (2H, m, CH_2_CH_3_), 1.68 (3H, s, CH_3_), 1.07 (3H, s, CH_3_), 1.01 (3H, s, CH_3_), 1.00 (3H, s, CH_3_), 0.99 (3H, t, *J* = 7.2 Hz, CH_2_ CH_3_), 0.85 (3H, s, CH_3_), 0.78 (3H, s, CH_3_); ^13^C NMR (150 MHz, CDCl_3_): *δ* 156.0 (O–C=O), 150.1 (C-20), 109.9 (C-29), 79.0 (C-3), 69.6, 66.4 (C-28), 55.3, 50.4, 48.8, 47.7, 46.6, 42.7, 40.9, 38.9, 38.7, 37.6, 37.1, 34.4, 34.2, 29.6, 29.5, 28.0, 27.4, 27.0, 25.2, 22.0, 20.8, 19.1, 18.3, 16.1, 16.0, 15.3, 14.8, 10.2; EIMS *m/z* 528 [M]^+^ (19), 189 (100).

28-*O*-Allyloxycarbonylbetulin (**11**) Yield 66 %; m.p. 91–94 °C; *R*_f_ 0.47 (chloroform/ethanol, 40:1, v/v); IR (KBr) *ν*_max_ 3405, 2962, 1737, 1457, 1270 cm^−1^; ^1^H NMR (600 MHz, CDCl_3_): *δ* 5.98 (1H, m, CH=CH_2_), 5.38 (1H, m, CH=CH_2_), 5.31 (1H, m, CH=CH_2_), 4.71 (1H, s, H-29), 4.66 (2H, m, OCH_2_), 4.61 (1H, s, H-29), 4.38 (1H, d, *J *= 10.8 Hz, H-28), 3.95 (1H, d, *J *= 10.8 Hz, H-28), 3.21 (1H, m, H-3), 2.46 (1H, m, H-19), 1.68 (3H, s, CH_3_), 1.06 (3H, s, CH_3_), 1.00 (3H, s, CH_3_), 0.99 (3H, s, CH_3_), 0.84 (3H, s, CH_3_), 0.78 (3H, s, CH_3_); ^13^C NMR (150 MHz, CDCl_3_): *δ* 155.6 (O–C=O), 150.1 (C-20), 131.7, 118.9, 109.9 (C-29), 78.9 (C-3), 68.5, 66.7 (C-28), 55.3, 50.4, 48.8, 47.7, 46.6, 42.7, 40.9, 38.9, 38.7, 37.6, 37.2, 34.4, 34.2, 29.7, 29.6, 28.0, 27.4, 27.0, 25.2, 20.8, 19.1, 18.3, 16.1, 16.0, 15.4, 14.8; EIMS *m/z* 496 [M]^+^ (14), 189 (100).

#### General procedure for the synthesis of derivatives **15**–**26**

To a solution of the appropriate monoester **3**–**14** (1 mmol) in dry dichloromethane (12 mL) was added pyridinium chloroformate (0.53 g, 2.48 mmol). The reaction was stirred at room temperature for 2 h and then diluted with ether (16 mL) and still was stirred for 10 min. The reaction mixture was filtered off through a layer of silica gel and washed with ether (5 mL). The filtrate was concentrated under reduced pressure and the residue was purified by silica gel column chromatography (chloroform/ethanol 40:1, v/v).

28-*O*-Propanoylbetulone (**15**) Yield 82 %; m.p. 65–67 °C; *R*_f_ 0.64 (chloroform/ethanol, 40:1, v/v); IR (KBr) *ν*_max_ 2945, 1733, 1705, 1462, 1187 cm^−1^; ^1^H NMR (600 MHz, CDCl_3_): *δ* 4.72 (1H, s, H-29), 4.62 (1H, s, H-29), 4.30 (1H, d, *J *= 10.8 Hz, H-28), 3.86 (1H, d, *J* = 10.8 Hz, H-28), 2.49 (1H, m, H-19), 2.37 (2H, q, *J* = 7.2 Hz, CH_2_CH_3_), 1.69 (3H, s, CH_3_), 1.18 (3H, t, *J* = 7.2 Hz, CH_2_ CH_3_), 1.10 (3H, s, CH_3_), 1.09 (3H, s, CH_3_), 1.05 (3H, s, CH_3_), 1.01 (3H, s, CH_3_), 0.96 (3H, s, CH_3_); ^13^C NMR (150 MHz, CDCl_3_ ): *δ* 218.0 (C=O), 174.9 (O–C=O), 150.1 (C-20), 109.9 (C-29), 62.5 (C-28), 55.0, 49.8, 48.8, 47.7, 47.4, 46.4, 42.8, 40.8, 39.6, 37.7, 36.9, 34.6, 34.2, 33.5, 29.8, 29.6, 27.7, 27.1, 26.6, 25.2, 21.3, 21.1, 19.6, 19.2, 15.9, 15.8, 14.7, 9.2; EIMS *m/z* 526 [M]^+^ (23), 189 (100).

28-*O*-(2-Propenoyl)betulone (**16**) Yield 62 %; m.p. 69–71 °C; *R*_f_ 0.62 (chloroform/ethanol, 40:1, v/v); IR (KBr) *ν*_max_ 2962, 1723, 1706, 1456, 1261 cm^−1^; ^1^H NMR (600 MHz, CDCl_3_): *δ* 6.34 (1H, m, CH=CH_2_), 6.06 (1H, m, CH=CH_2_), 5.75 (1H, m, CH=CH_2_), 4.63 (1H, s, H-29), 4.53 (1H, s, H-29), 4.30 (1H, d, *J* = 10.8 Hz, H-28), 3.86 (1H, d, *J* = 10.8 Hz, H-28), 2.40 (1H, m, H-19), 1.65 (3H, s, CH_3_), 1.01 (3H, s, CH_3_), 1.00 (3H, s, CH_3_), 0.96 (3H, s, CH_3_), 0.92 (3H, s, CH_3_), 0.87 (3H, s, CH_3_); ^13^C NMR (150 MHz, CDCl_3_): *δ* 217.0 (C=O), 165.6 (O–C=O), 149.0 (C-20), 129.5, 127.6, 108.9 (C-29), 61.8 (C-28), 53.9, 48.7, 47.7, 46.7, 46.3, 45.5, 41.8, 39.8, 38.6, 36.7, 35.9, 33.5, 33.1, 32.4, 28.7, 28.6, 26.1, 25.5, 24.2, 20.3, 20.0, 18.6, 18.1, 14.9, 14.8, 13.7; EIMS *m/z* 495 [M]^+^ (27), 203 (100).

28-*O*-Propynoylbetulone (**17**) Yield 78 %; m.p. 93–96 °C; *R*_f_ 0.65 (chloroform/ethanol, 40:1, v/v); IR (KBr) *ν*_max_ 3301, 2948, 2117, 1712, 1708, 1225 cm^−1^; ^1^H NMR (600 MHz, CDCl_3_): *δ* 4.69 (1H, s, H-29), 4.60 (1H, s, H-29), 4.38 (1H, d, *J* = 10.8 Hz, H-28), 3.99 (1H, d, *J* = 10.8 Hz, H-28), 2.89 (1H, s, C≡CH), 2.48 (1H, m, H-19), 1.68 (3H, s, CH_3_), 1.07 (3H, s, CH_3_), 1.06 (3H, s, CH_3_), 1.03 (3H, s, CH_3_), 0.88 (3H, s, CH_3_), 0.80 (3H, s, CH_3_); ^13^C NMR (150 MHz, CDCl_3_): *δ* 217.9 (C=O), 153.2 (O-C=O), 149.8 (C-20), 110.0 (C-29), 74.8, 74.6, 64.8 (C-28), 55.0, 49.7, 48.8, 47.6, 47.3, 46.4, 42.8, 40.8, 39.6, 37.8, 36.9, 34.4, 34.1, 33.5, 29.6, 29.5, 27.0, 26.6, 25.2, 21.3, 21.0, 19.6, 19.1, 15.9, 15.8, 14.7; EIMS *m/z* 492 [M]^+^ (28), 203 (100).

28-*O*-(3-Cyclopropyl-2-propynoyl)betulone (**18**) Yield 83 %; m.p. 93–97 °C; *R*_f_ 0.65 (chloroform/ethanol, 40:1, v/v); IR (KBr) *ν*_max_ 2947, 2232, 1711, 1705, 1457, 1249 cm^−1^; ^1^H NMR (600 MHz, CDCl_3_): *δ* 4.71 (1H, s, H-29), 4.62 (1H, s, H-29), 4.35 (1H, d, *J* = 10.8 Hz, H-28), 3.96 (1H, d, *J* = 10.8 Hz, H-28), 2.43 (1H, m, H-19), 1.68 (3H, s, CH_3_), 1.09 (3H, s, CH_3_), 1.08 (3H, s, CH_3_), 1.05 (3H, s, CH_3_), 1.00 (3H, s, CH_3_), 0.97–0.96 (5H, m, CH, CH_2_), 0.95 (3H, s, CH_3_); ^13^C NMR (150 MHz, CDCl_3_): *δ* 218.5 (C=O), 155.0 (O–C=O), 150.5 (C-20), 110.5 (C-29), 93.9, 69.1, 64.6 (C-28), 55.5, 50.3, 49.3, 48.2, 47.9, 46.9, 43.3, 41.4, 40.2, 38.3, 37.4, 35.1, 34.7, 34.0, 30.2, 30.1, 27.6, 27.1, 25.8, 21.9, 21.6, 20.2, 19.7, 16.5, 16.4, 15.2, 9.4 1.3, -0.4; EIMS *m/z* 533 [M]^+^ (14), 93 (100).

28-*O*-Phenylpropynoylbetulone (**19**) Yield 82 %; m.p. 82–85 °C; *R*_f_ 0.64 (chloroform/ethanol, 40:1, v/v); IR (KBr) *ν*_max_ 2945, 2223, 1722, 1707, 1457, 1187 cm^−1^; ^1^H NMR (600 MHz, CDCl_3_): *δ* 7.60–7.37 (5H, m, Ar-H), 4.71 (1H, s, H-29), 4.61 (1H, s, H-29), 4.43 (1H, d, *J* = 10.8 Hz, H-28), 4.04 (1H, d, *J* = 10.8 Hz, H-28), 2.41 (1H, m, H-19), 1.69 (3H, s, CH_3_), 1.09 (3H, s, CH_3_), 1.07 (3H, s, CH_3_), 1.03 (3H, s, CH_3_), 1.00 (3H, s, CH_3_), 0.94 (3H, s, CH_3_); ^13^C NMR (150 MHz, CDCl_3_): *δ* 218.0 (C=O), 154.7 (O–C=O), 149.9 (C-20), 132.9, 130.6, 128.5, 110.0 (C-29), 86.3, 80.7, 64.5 (C-28), 54.9, 49.7, 48.8, 47.6, 47.3, 46.4, 42.8, 40.8, 39.6, 37.8, 36.8, 34.5, 34.1, 33.5, 29.6, 29.5, 27.0, 26.6, 25.2, 21.3, 21.0, 19.6, 19.1, 15.9, 15.8, 14.7; EIMS *m/z* 569 [M]^+^ (16), 129 (100).

28-*O*-(2-Butenoyl)betulone (**20**) Yield 81 %; m.p. 129–131 °C; R_f_ 0.63 (chloroform/ethanol, 40:1, v/v); IR (KBr) *ν*_max_ 2944, 1719, 1708, 1457, 1179 cm^−1^; ^1^H NMR (600 MHz, CDCl_3_): *δ* 7.00 (1H, m, CH=CHCH_3_), 5.89 (1H, m, CH=CHCH_3_), 4.72 (1H, s, H-29), 4.62 (1H, s, H-29), 4.34 (1H, d, *J* = 10.8 Hz, H-28), 3.93 (1H, d, *J* = 10.8 Hz, H-28), 2.42 (1H, m, H-19), 1.92 (3H, m, CH=CHCH_3_), 1.69 (3H, s, CH_3_), 1.10 (3H, s, CH_3_), 1.09 (3H, s, CH_3_), 1.07 (3H, s, CH_3_), 1.01 (3H, s, CH_3_), 0.96 (3H, s, CH_3_); ^13^C NMR (150 MHz, CDCl_3_): *δ* 218.1 (C=O), 167.0 (O–C=O), 150.1 (C-20), 144.5, 122.8, 109.9 (C-29), 62.4 (C-28), 55.0, 49.8, 48.8, 47.7, 47.4, 46.5, 42.8, 40.8, 39.6, 37.7, 36.8, 34.6, 34.2, 33.5, 29.8, 29.6, 27.1, 26.6, 25.2, 21.3, 21.1, 19.6, 18.0, 15.9, 15.8, 14.7, 3.6; EIMS *m/z* 510 [M]^+^ (14), 189 (100).

28-*O*-(2-Butynoyl)betulone (**21**) Yield 78 %; m.p. 92–94 °C; *R*_f_ 0.66 (chloroform/ethanol, 40:1, v/v); IR (KBr) *ν*_max_ 2960, 2245, 1742, 1707, 1458, 1260 cm^−1^; ^1^H NMR (600 MHz, CDCl_3_): *δ* 4.69 (1H, s, H-29), 4.59 (1H, s, H-29), 4.34 (1H, d, *J* = 10.8 Hz, H-28), 3.94 (1H, d, *J* = 10.8 Hz, H-28), 2.44 (1H, m, H-19), 1.99 (3H, s, C≡CCH_3_), 1.68 (3H, s, CH_3_), 1.07 (3H, s, CH_3_), 1.06 (3H, s, CH_3_), 1.03 (3H, s, CH_3_), 0.99 (3H, s, CH_3_), 0.93 (3H, s, CH_3_); ^13^C NMR (150 MHz, CDCl_3_): *δ* 218.0 (C=O), 154.4 (O–C=O), 149.9 (C-20), 110.0 (C-29), 85.6, 72.5, 64.2 (C-28), 55.0, 49.7, 48.8, 47.6, 47.3, 46.4, 42.7, 40.8, 39.6, 37.7, 36.8, 34.5, 34.1, 33.5, 29.6, 29.5, 27.0, 26.6, 25.2, 21.3, 21.0, 19.6, 19.1, 15.9, 15.8, 14.7, 3.8; EIMS *m/z* 507 [M]^+^ (29), 422 (100).

28-*O*-Propoxycarbonylbetulone (**22**) Yield 78 %; m.p. 76–78 °C; *R*_f_ 0.63 (chloroform/ethanol, 40:1, v/v); IR (KBr) *ν*_max_ 2963, 1741, 1700, 1458, 1256 cm^−1^; ^1^H NMR (600 MHz, CDCl_3_): *δ* 4.72 (1H, s, H-29), 4.62 (1H, s, H-29), 4.37 (1H, d, *J *= 10.8 Hz, H-28), 4.13 (2H, t, *J* = 6.6 Hz, OCH_2_), 3.95 (1H, d, *J* = 10.8 Hz, H-28), 2.45 (1H, m, H-19), 1.74 (2H, m, CH_2_CH_3_), 1.68 (3H, s, CH_3_), 1.10 (3H, s, CH_3_), 1.09 (3H, s, CH_3_), 1.05 (3H, s, CH_3_), 1.01 (3H, s, CH_3_), 0.99 (3H, t, *J* = 7.2 Hz, CH_2_ CH_3_), 0.96 (s, CH_3_, 3H); ^13^C NMR (150 MHz, CDCl_3_): *δ* 218.0 (C=O), 156.0 (O–C=O), 150.0 (C-20), 109.9 (C-29), 69.6, 66.4 (C-28), 55.0, 49.7, 48.8, 47.7, 46.6, 42.8, 40.8, 39.6, 37.7, 36.9, 34.4, 34.2, 33.5, 29.6, 29.5, 27.1, 26.6, 25.6, 25.2, 22.1, 21.3, 21.1, 19.6, 19.1, 15.9, 15.8, 14.7, 10.2; EIMS *m/z* 526 [M]^+^ (15), 422 (100).

28-*O*-Allyloxycarbonylbetulone (**23**) Yield 72 %; m.p. 123–125 °C; *R*_f_ 0.66 (chloroform/ethanol, 40:1, v/v); IR (KBr) *ν*_max_ 2945, 1741, 1710, 1457, 1248 cm^−1^; ^1^H NMR (600 MHz, CDCl_3_ ): *δ* 5.98 (1H, m, CH=CH_2_), 5.40 (1H, m, CH=CH_2_), 5.31 (1H, m, CH=CH_2_), 4.72 (1H, s, H-29), 4.66 (2H, m, OCH_2_), 4.62 (1H, s, H-29), 4.38 (1H, d, *J *= 10.8 Hz, H-28), 3.96 (1H, d, *J *= 10.8 Hz, H-28), 2.44 (1H, m, H-19), 1.68 (3H, s, CH_3_), 1.10 (3H, s, CH_3_), 1.09 (3H, s, CH_3_), 1.08 (3H, s, CH_3_), 1.05 (3H, s, CH_3_), 0.96 (3H, s, CH_3_);^13^C NMR (150 MHz, CDCl_3_ ): *δ* 218.0 (C=O), 155.6 (O–C=O), 150.0 (C-20), 131.7, 119.0, 109.9 (C-29), 68.5, 66.6 (C-28), 55.0, 53.4, 49.7, 48.8, 47.7, 47.4, 46.6, 42.8, 40.8, 39.6, 37.7, 36.9, 34.4, 34.2, 33.5, 29.6, 29.5, 27.0, 26.6, 25.2, 21.3, 21.1, 19.6, 15.9, 15.8, 14.7; EIMS *m/z* 524 [M]^+^ (19), 422 (100).

28-*O*-Propargyloxycarbonylbetulone (**24**) Yield 73 %; m.p. 173–175 °C; *R*_f_ 0.64 (chloroform/ethanol, 40:1, v/v); IR (KBr) *ν*_max_ 3245, 2944, 2131, 1721, 1706, 1452, 1269 cm^−1^; ^1^H NMR (600 MHz, CDCl_3_): *δ* 4.74 (2H, d, *J* = 2.4 Hz, OCH_2_), 4.69 (1H, s, H-29), 4.60 (1H, s, H-29), 4.38 (1H, d, *J* = 10.8 Hz, H-28), 3.96 (1H, d, *J* = 10.8 Hz, H-28), 2.54 (1H, t, *J* = 2.4 Hz, C≡CH), 2.41 (1H, m, H-19), 1.68 (3H, s, CH_3_), 1.09 (3H, s, CH_3_), 1.07 (3H, s, CH_3_), 1.03 (3H, s, CH_3_), 0.99 (3H, s, CH_3_), 0.93 (3H, s, CH_3_); ^13^C NMR (150 MHz, CDCl_3_ ): *δ* 218.0 (C=O), 155.1 (O–C=O), 149.9 (C-20), 110.0 (C-29), 76.8, 75.6, 75.4, 67.2 (C-28), 55.2, 54.9, 49.7, 48.7, 47.6, 46.5, 42.8, 40.8, 39.6, 37.7, 36.8, 34.3, 34.1, 33.4, 29.5, 29.4, 26.9, 26.6, 25.2, 21.3, 21.0, 19.6, 19.1, 15.9, 15.8, 14.7; EIMS *m/z* 523 [M]^+^ (24), 189 (100).

28-*O*-(3-Butynyloxycarbonyl)betulone (**25**) Yield 77 %; m.p. 81–84 °C; *R*_f_ 0.63 (chloroform/ethanol, 40:1, v/v); IR (KBr) *ν*_max_ 3310, 2948, 2360, 1744, 1705, 1458, 1248 cm^−1^; ^1^H NMR (600 MHz, CDCl_3_): *δ* 4.69 (1H, s, H-29), 4.59 (1H, s, H-29), 4.36 (1H, d, *J* = 10.8 Hz, H-28), 4.25 (2H, t, *J* = 7.2 Hz, OCH_2_), 3.94 (1H, d, *J* = 10.8 Hz, H-28), 2.60 (2H, m, OCH_2_ CH_2_), 2.41 (1H, m, H-19), 2.03 (1H, t, *J* = 2.4 Hz, C≡CH), 1.68 (3H, s, CH_3_), 1.08 (3H, s, CH_3_), 1.07 (3H, s, CH_3_), 1.03 (3H, s, CH_3_), 0.98 (3H, s, CH_3_), 0.93 (3H, s, CH_3_); ^13^C NMR (150 MHz, CDCl_3_): *δ* 218.2 (C=O), 155.5 (O–C=O), 149.9 (C-20), 110.0 (C-29), 79.4, 76.8, 70.2, 66.7 (C-28), 65.3, 54.9, 49.6, 48.7, 47.6, 47.3, 46.5, 42.7, 40.8, 39.6, 37.6, 36.8, 34.3, 34.1, 33.4, 29.5, 29.4, 26.9, 26.5, 25.1, 21.2, 21.0, 19.6, 19.0, 15.9, 15.8, 14.6; EIMS *m/z* 537 [M]^+^ (14), 189 (100).

28-*O*-(2-Butynyloxycarbonyl)betulone (**26**) Yield 75 %; m.p. 150–152 °C; *R*_f_ 0.59 (chloroform/ethanol, 40:1, v/v); IR (KBr) *ν*_max_ 2956, 2231, 1742, 1704, 1458, 1255 cm^−1^; ^1^H NMR (600 MHz, CDCl_3_): *δ* 4.71 (2H, q, *J* = 2.4 Hz, OCH_2_), 4.69 (1H, s, H-29), 4.59 (1H, s, H-29), 4.36 (1H, d, *J* = 10.8 Hz, H-28), 3.93 (1H, d, *J* = 10.8 Hz, H-28), 2.42 (1H, m, H-19), 1.87 (3H, t, *J* = 2.4 Hz, C≡CCH_3_), 1.68 (3H, s, CH_3_), 1.08 (3H, s, CH_3_), 1.07 (3H, s, CH_3_), 1.03 (3H, s, CH_3_), 0.98 (3H, s, CH_3_), 0.93 (3H, s, CH_3_); ^13^C NMR (150 MHz, CDCl_3_): *δ* 218.2 (C=O), 155.2 (O–C=O), 149.9 (C-20), 110.0 (C-29), 84.1, 76.7, 72.6, 66.9 (C-28), 56.1, 54.9, 49.6, 48.7, 47.6, 47.3, 46.5, 42.7, 40.8, 39.6, 37.7, 36.8, 34.3, 33.4, 29.5, 29.4, 26.9, 26.5, 25.1, 21.2, 21.0, 19.6, 19.1, 15.9, 15.8, 14.6, 3.7; EIMS *m/z* 537 [M]^+^ (14), 189 (100).

### Antiproliferative Assay in vitro

#### Cells

The targeted compounds were evaluated for their cytotoxicity towards the cancer cell lines including T47D (human breast cancer), CCRF/CEM (human leukemia), SW707 (human colorectal), HL-60 (human promyelocytic leukemia), P388 (mouse leukemia), as well as BALB3T3 normal mouse fibroblasts cell line. The tested cell lines were obtained from the American Type Culture Collection (Rockville, Maryland, USA) and maintained at the Cell Culture Collection of the Institute of Immunology and Experimental Therapy (Wrocław, Poland). The cells were seeded in 96-well plates (Sarstedt, USA) at a density of 10^4^ cells per well in 100 µL of culture medium overnight. The cancer cell lines CCRF/CEM (human leukemia) and P388 (mouse leukemia) were cultured in RPMI 1640 medium (Gibco, Scotland, UK) supplemented with 2 mM glutamine (Sigma-Aldrich, Chemie GmbH, Steinheim, Germany) and 10 % fetal calf serum FBS (Sigma-Aldrich, Chemie GmbH, Steinheim, Germany). The cancer cell lines SW707 (human colorectal), T47D (human breast cancer) and HL-60 (human promyelocytic leukemia) were cultured in mixture of RPMI 1640 and Opti-MEM (1:1) medium (both from Gibco, Scotland, UK) supplemented with 2 mM glutamine (Sigma-Aldrich, Chemie GmbH, Steinheim, Germany) and 5 % or 20 % fetal calf serum FBS (Sigma-Aldrich, Chemie GmbH, Steinheim, Germany). The normal mouse fibroblasts BALB3T3 was cultured in Dulbecco’s modified Eagle’s medium (Sigma-Aldrich, Chemie GmbH, Steinheim, Germany) supplemented with 2 mM glutamine (Sigma-Aldrich, Chemie GmbH, Steinheim, Germany) and 10 % fetal calf serum FBS (Sigma-Aldrich, Chemie GmbH, Steinheim, Germany). The all culture media were supplemented with streptomycin (100 µg/mL) and penicillin (100 U/mL) (both antibiotics from Polfa, Tarchomin, Poland). The cell cultures were maintained at 37 °C in humid atmosphere saturated with 5 % CO_2_.

#### SRB assay

This technique as first was described by Skehan et al. in 1990. The SRB assay was performed after 96 h exposure of the cultured cells to varying concentrations (ranging from 1 to 100 µg/mL) of the tested substances. The reported derivatives as well as betulin **1** were dissolved in 10 % dimethyl sulfoxide (DMSO) to concentration of 1 mg/mL, and next diluted in culture medium to reach the required concentrations. DMSO as a solvent did not exert any inhibitory effect on cell proliferation. The cells fastened to the plastic were fixed mildly layering cold 50 % TCA (trichloroacetic acid, Aldrich-Chemie, Germany) on the top of the culture medium in each well. The plates were incubated at 4 °C for 1 h and then washed five times with tap water. The cells fixed with TCA were stained with 0.4 % sulforhodamine B (SRB, Sigma, Germany) dissolved in 1 % acetic acid (POCH, Gliwice, Poland) for 30 min. The unbound dye was removed by rinsing four times with 1 % acetic acid. The protein-bound dye was extracted with 10 mM unbuffered tris base (POCH, Gliwice, Poland) and then determined the optical density at 540 nm in a computer-interfaced, 96-well microtiter plate reader Multiscan RC photometer (Labsystems, Helsinki, Finland).

The compounds in given concentration were examined in triplicates in each experiment which was repeated 3–5 times. The results of cytotoxic activity in vitro were expressed as an IC_50_ in µg/mL.

#### MTT assay

The MTT assay was used for the cytotoxicity screening against leukemia cells growing in suspension culture. This assay was performed after 96 h of the leukemia cells to varying concentrations (ranging from 1 to 100 µg/mL) of the tested substances. The derivatives of betulin were dissolved in 10 % DMSO to concentration of 1 mg/mL, and next diluted in culture medium to reach the required concentrations. DMSO as a solvent did not exert any inhibitory effect on cell proliferation. During the last 3–4 h of incubation 20 µl of MTT solution were added to each well (MTT: 3-(4,5-dimethylthiazol-2-yl)-2,5-diphenyl tetrazolium bromide; stock solution: 5 mg/mL). The pale yellow MTT is reduced to a navy blue formazan in the mitochondria of living cells. At the end of incubation time, 80 µL of the lysing mixture was added to each well (lysing mixture: 225 mL dimethylformamide, 67.5 g sodium dodecyl sulfate and 275 mL of distilled water). After 24 h are formed the crystals of formazan, which are insoluble in aqueous solutions. The formazan crystals had been dissolved. The optical densities were read on a Multiskan RC photometer at 570 nm wavelength.

The compounds in given concentration were examined in triplicates in each experiment which was repeated 3–5 times. The results of cytotoxic activity in vitro were expressed as an IC_50_ in µg/mL.

#### In silico study

The physicochemical properties of obtained compounds such as lipophilicity (cLogP), molecular mass (M), topological polar surface area (tPSA), hydrogen bond donors (HBD) and hydrogen bond acceptors (HBA) were calculated using the ACD/Labs software.

## Results and discussion

### Chemistry

**Scheme 1:**
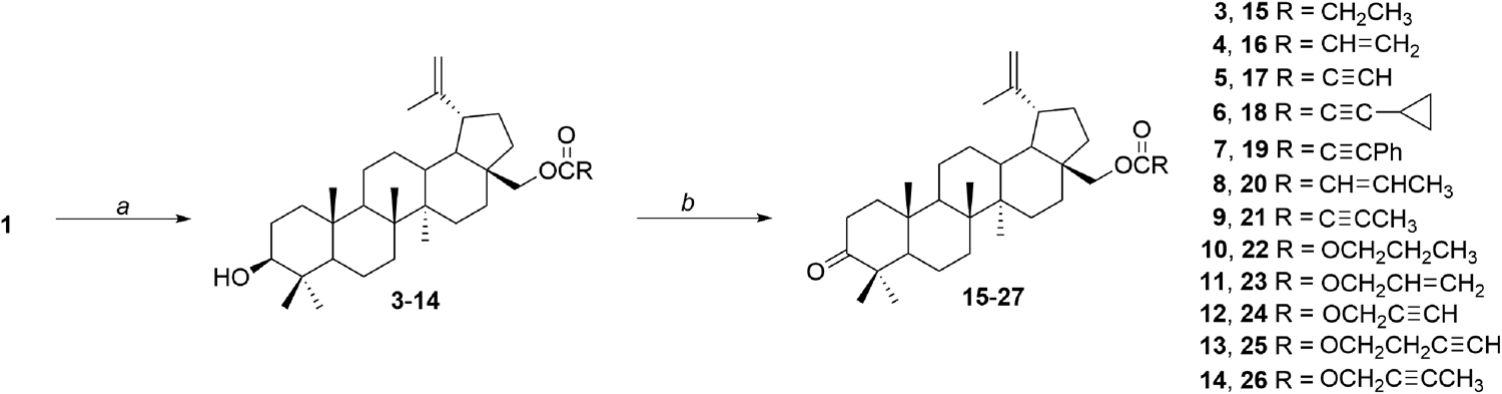
Synthesis of derivatives **3**–**26**. *Reagents and conditions*: *a* RCOOH, CH_2_Cl_2_, DCC, DMAP, rt, 24 h or ROC(O)Cl, benzene, pyridine, rt, 24 h; *b* PCC, CH_2_Cl_2_, rt, 2 h

The synthesis of betulin derivatives **3**–**14** was accomplished starting with betulin **1**, which was isolated from birch bark of *Betula verrucosa*. The crude compound **1** was purified by flash-chromatography using a mixture of dichloromethane and ethanol as an eluent. Derivatives **3**–**14** were obtained according to our published procedures (Boryczka et al., 2013a, b). The esterification reactions of betulin **1** with carboxylic acids or chloroformates were performed with 49–86 % yields. The resulting monoesters **3**–**14** were oxidized with pyridinium chloroformate in dry dichloromethane to the derivatives of betulone **15**–**26** in 62–83 % yields. The synthesis of compounds **3**–**26** was presented via Scheme 1. All compounds were purified by column chromatography using the mixture of chloroform and ethanol. The chemical structures of new derivatives were determined on the basis of their ^1^H-NMR, ^13^C-NMR, IR and MS spectra.

### Cytotoxic activity

The newly compounds and betulin **1** were tested in vitro for their antitumor activity towards the following human cancer cell lines: T47D (human breast cancer), CCRF/CEM (human leukemia), SW707 (human colorectal), HL-60 (human promyelocytic leukemia) and murine leukemia P388 as well as BALB3T3 normal mouse fibroblasts cell line. In this study, cisplatin was used as a reference anticancer agent. The IC_50_ values (µg/mL) of the betulin derivatives are reported in Table 1 and Table 2. The compounds **3**–**9**, containing acyloxy group at the C-28 position, had IC_50_ values in the range of 0.02–49.0 µg/mL against the tested cell lines. In the series of the monoesters **3**–**9** the cytotoxic activity depends on the type of bond in the substituent at the C-28 position. The structure–activity relationships observed in the monoesters **3**–**9** indicates that rank order of the antiproliferative activity against the HL-60 cancer cell line, is as follows: propynoyl > propenoyl > 2-butynoyl > propanoyl > 3-cyclopropyl-2-propynoyl. Oxidation of the 3-hydroxyl group in compounds **4**, **6** and **10** to a carbonyl group led to an increase of activity against the HL-60 cancer cell line. The compounds **5** and **17** possessed the most potent activity, with IC_50_ value 0.3 µg/mL towards the human HL-60 cancer cell line. Moreover, these compounds exhibited the same antiproliferative activity as cisplatin. The betulones **24**–**26** with the acetylenic formate group at the C-28 position showed loss of activity against the human colorectal cell line (SW707) and normal mouse fibroblast cell line (BALB3T3). The rank order of the cytotoxic activity of the compounds **24**–**26** against T47D and CCRF/CEM cell lines, according to the nature of the formate substituent is as follows: propargyl > 3-butynyl > 2-butynyl.

**Table 1:** Cytotoxic activity (IC_50_) of betulin **1**, derivatives of betulin and cisplatin as a reference compound against the four cancer cell lines

Compound	Cytotoxic activity IC_50_ [μg/mL]				
	Human			Murine	
	T47D	CCRF/CEM	SW707	P388	BALB3T3
**Betulin 1**	32.4 ± 10.7	10.9 ± 5.5	22.9 ± 15.4	5.5 ± 3.3	47.3 ± 7.9
**3** ^a^	12.1 ± 4.4	8.1 ± 0.9	29.2 ± 24.4	3.3 ± 0.8	32.3 ± 23.0
**4**	7.1 ± 2.7	19.6 ± 9.5	29.6 ± 4.2	9.9 ± 5.9	24.3 ± 6.9
**5** ^a^	9.1 ± 1.9	0.02 ± 0.001	14.9 ± 3.3	0.4 ± 0.1	0.3 ± 0.05
**6**	33.6 ± 6.3	16.5 ± 8.5	Neg	8.4 ± 9.2	Neg
**7** ^a^	Neg	49.0 ± 9.8	Neg	Neg	Neg
**8**	29.9 ± 9.3	28.0 ± 21.6	32.0 ± 4.2	5.1 ± 4.9	25.6 ± 1.8
**9**	16.7 ± 5.5	2.1 ± 0.3	24.3 ± 8.8	2.9 ± 1.4	17.4 ± 1.1
**19**	Neg	Neg	Neg	Neg	Neg
**24**	23.3 ± 3.5	18.7 ± 6.6	Neg	30.3 ± 11.8	Neg
**25**	29.8 ± 10.7	25.2 ± 4.6	Neg	3.8 ± 1.9	Neg
**26**	79.2 ± 5.2	47.6 ± 5.7	Neg	18.3 ± 12.9	Neg
**Cisplatin**	3.1 ± 1.0	2.0 ± 0.5	2.2 ± 0.5	0.5 ± 0.3	2.7 ± 0.3

*Neg* negative in the concentration used

^a^ Boryczka et al. (2013a, b)

**Table 2:** Cytotoxic activity (IC_50_) of betulin **1**, derivatives of betulin and cisplatin as a reference compound against the HL-60 cancer cell line. The parameters determined by computational methods such as lipophilicity (cLogP), molecular mass (M), topological polar surface area (tPSA), hydrogen bond donors (HBD) and hydrogen bond acceptors (HBA)

Compound	IC_50_ [μg/mL]	cLogP	M	tPSA [Å]	HBD	HBA
**Betulin 1**	7.2 ± 0.5	6.63	442.71	40.46	2	2
**3**	29.3 ± 3.4	8.42	498.78	46.53	1	3
**4**	18.8 ± 4.8	8.23	496.76	46.53	1	3
**5**	0.3 ± 0.004	7.76	494.75	46.53	1	3
**6**	44.8 ± 10.9	8.85	534.81	46.53	1	3
**9**	24.5 ± 1.9	8.31	508.77	46.53	1	3
**10**	26.8 ± 2.5	8.39	528.80	55.76	1	4
**11**	14.9 ± 3.9	8.01	526.79	55.76	1	4
**12**	3.7 ± 0.5	7.64	524.77	55.76	1	4
**15**	33.7 ± 5.5	8.13	496.76	43.37	0	3
**16**	5.0 ± 1.3	8.07	494.75	43.37	0	3
**17**	0.3 ± 0.06	7.66	492.73	43.37	0	3
**18**	33.9 ± 5.5	8.61	532.79	43.37	0	3
**21**	29.5 ± 5.4	8.39	506.76	43.37	0	3
**22**	21.4 ± 2.1	8.23	526.79	52.60	0	4
**23**	28.4 ± 1.6	8.05	524.77	52.60	0	4
**24**	11.6 ± 4.9	7.68	522.76	52.60	0	4
**Cisplatin**	0.3 ± 0.08	—	—	—	—	—

The in silico study of tested compounds was performed by determination of Lipinski’s rule of five and tPSA. The number of HBA and HBD significantly modulates the size of the polar surface area of molecule. The increase of the HBA number leads to higher affinity of the derivatives of betulone for P-glycoprotein. The most of betulin derivatives exhibited high values of molecular mass (M > 500) and lipophilicity (cLogP > 5). However, the tPSA of all compounds is less than 140 Å, what determines high oral bioavailability (Abd El-Karim et al., 2015).

In conclusion, the newly derivatives of betulin and betulone has been synthesized and characterized by spectroscopic analyses. Derivatives of betulin were tested for their antiproliferative activity against the five cancer cell lines. Several compounds exhibited a better cytotoxic effect than betulin **1**. The most active derivatives **5** and **17** were 24-fold potent than betulin **1** against the human promyelocytic leukemia cell line (HL-60).
